# The Psychometric Properties of the Student–Teacher Relationship Measure for Omani Grade 7–11 Students

**DOI:** 10.3389/fpsyg.2019.02283

**Published:** 2019-10-11

**Authors:** Said Aldhafri, Amal Alhadabi

**Affiliations:** ^1^Department of Psychology, College of Education, Sultan Qaboos University, Muscat, Oman; ^2^Social Observatory Research Program, The Research Council, Muscat, Oman; ^3^School of Foundations, Leadership and Administration, College of Education, Health and Human Services, Kent State University, Kent, OH, United States; ^4^Center for Teaching and Learning, Kent State University, Kent, OH, United States

**Keywords:** teacher–student relationship, measure development, Oman, Arab, teaching

## Abstract

Development and validation of a 25-item Student–Teacher Relationship Measure is described. It is a self-report measure estimating students’ perceptions of their relationship with teachers. The study was applied among adolescents in grades 7–11 in Oman. The measure was administered in Arabic. In Study 1, findings from exploratory factor analysis for 1,035 students indicated the presence of a 2-factor model (academic relation and social relation). In study 2, the confirmatory factor analysis results of 1,099 students supported study 1 results. High internal consistency was acquired. STRM was regarded as a reliable and potentially valid measure of the quality of student–teacher relationships.

## Introduction

The main goal of education is to help students improve in two aspects of their lives, namely academic and social facets, which are greatly influenced by the amount and the quality of their engagement and comfort in the school environment (Gregorg et al., 2014). There are several factors in the school environment that have an impact on education, such as administration, curriculum, school norms, classroom atmosphere, teacher-student relationship, teachers, and students themselves. Undoubtedly, one of the most important factors is the teacher–student relationship (STR), which can shape students’ learning and school engagement, positively or negatively, in three dimensions – behavioral, cognitive, and emotional engagement (Gregorg et al., 2014). Moreover, a good STR can enhance positive student development regarding the motivation, high levels of engagement, academic achievement and social-emotional adjustment (Northup, 2011). To shape the quality of the STR, teachers play a great role through two processes – interaction and instruction. The positive STR enhances establishing a productive classroom environment, which in turn, promotes an effective learning mode and enhance academic performance. Productive classroom environment also facilitates a supportive classroom community in which students’ needs (e.g., physical, social, cognitive, and affective needs) are met (Merritt, 2018).

A comprehensive review of literature related to STR has emphasized multiple points. First, this construct has been studied by several theoretical orientations (e.g., Attachment Theory, Sociocultural Theory, Ecologcal Theory, Social Cognitive Theory, and Developmental Systems Theory). Correspondingly, multiple scales assess STR, which have diverse factorial structures. For example, three dimensions were supported (e.g., conflict, closeness, and dependency; Pianta, 2001), as well as four dimensions (e.g., trust, respect, communication, and discipline; Riddle, 2003). Furthermore, many approaches have existed in measuring this construct (i.e., assessing teachers’ perspectives, students’ perspectives, observations, and case studies). Teachers’ and students’ views on the STR differ according to a set of variables and conditions. For example, according to teachers, a study conducted by Poulou (2017), which included a sample of 92 preschool Greek teachers, highlighted that teachers’ perception toward a positive STR is significantly correlated with their perceptions toward their emotional intelligence (EI) level and the comfort to apply social and emotional learning (SEL) practices. In other words, teachers who viewed themselves as they have high score of EI and feel comfortable to use SEL have positive perception toward the relationship with their students. Thus, many scales measure the relations from teachers’ perspective (Pianta, 2001; Ang, 2005).

The current study was more interested in assessing students’ perceptions. Students-related variables might influence their perceptions about STR (e.g., age, grade, and gender; Lee, 2012). The literature has articulated that majority of well-established scales examined the perceptions of very young children, particularly kindergarten and elementary school students (e.g., Emotional Quality Scale of the Relatedness Questionnaire, Quality of Teacher-Student Relationship Scale, and Inventory of Teacher–Student Relationship; Lynch and Cicchetti, 1997; Davis, 2001; Murray and Zvoch, 2011). Consequently, little was known about middle school students’ relations with their teachers (Saft and Pianta, 2001) and seemingly even less about high school age students (Northup, 2011). Differences do exist in students’ developmental characteristics and psychological needs between adolescents in middle/high schools and younger students in kindergarten/elementary schools, necessitating the development of scale that tailored for middle/high schools’ students. Majority of pervious scales examined STR among students in the individualistic culture (Hofstede, 2001), thus little is known about collectivistic cultures including Middle East. Most critically, no standardized Arabic scale measuring this relationship has been developed. Therefore, it is necessary to measure the relationship between teachers and students from different perspectives.

Moreover, the influences of STR varies across subjects and students’ gender. At the subject level (e.g., STEM subjects), this relationship determines how students deal with difficult learning tasks in math and science (Mikk et al., 2016). In particular, the relationship with science teacher has a critical role among rural female students, owing to females had a weaker science identity relative to boys (Hill et al., 2018). Thus, positive relations with science teachers might empower female’s academic achievement and enrollment in STEM majors, particularly in the Middle East. Hence, the aim of this study was twofold, which includes: (1) Developing a student-teacher relationship measure as perceived by 7–11 grade female students, in which they rated their relations with science teachers and (2) Examining its’ psychometric properties using exploratory and confirmatory factor analyses.

### Literature Review

#### Student–Teacher Relationship (STR)

Much recently produced literature and educational research focus on investigating factors that affect students’ learning, adjustment and outcomes in a positive way, both academically such as high achievement scores (Lee, 2012), academic engagement (Gregorg et al., 2014), motivation to learn (Alhadabi, 2013), and self-concept (Alrajhi and Aldhafri, 2015) and socially such as personal and school adjustment (Baker, 2006), reducing misbehavior (Baker et al., 2008), and gaining social skills (Berry and O’Connor, 2010). Many studies have stated that the STR is one of these factors with the greatest influence on students’ learning, and their academic and social lives (Northup, 2011). In this research, a STR is defined as social and academic relations between a teacher and students. It is greatly influenced by a teacher’s personal characteristics (e.g., the level of caring, the ability to promote trust, and create a safe learning environment); as well as instructional characteristics (e.g., considering the differences in students’ learning styles, applying management styles, and motivating students). These all contribute to the formation of positive students’ outcomes (i.e., cognitive, behavioral and social outcomes).

Regarding cognitive outcomes, a constructive STR fosters a positive development, including high levels of engagement (Roorda et al., 2011), productive school attitudes, values and goals (e.g., high expectations, interests, intrinsic motivation, willingness to succeed, satisfaction with school, and self-efficacy; Wentzel, 2002). Thus, these relations resulted in high performance (Baker et al., 2008; Rodriguez, 2008; Lee, 2012) and high self-regulated learning (Deiro, 2003). On the other hand, negative relations are associated with low grades (DiLalla et al., 2004), school drop-out (Brewster and Bowen, 2004) and dismissive academic behaviors (Fredricks et al., 2004).

Related to behavioral outcomes, STRs have a constant effect on students’ behavioral engagement which is defined as “adolescents’ effort, attention and persistence during the initiation and execution of learning activities” (Skinner et al., 2008; as cited in Engels et al., 2016, p. 1192). Engels et al. (2016) investigated the transactional links between positive and negative teacher-adolescent relationships and their behavioral engagements on the learning tasks. This longitudinal study included 1,116 students who were from 7th to 11th grade. It was found that students who showed a high level of behavioral engagement had positive relations with their teachers during the 3 years. On the other hand, those who had negative STR revealed a low level of behavioral engagement over time. Additionally, the perceived positive relations have a great influence on students’ academic engagement (Bakhshaee and Hejazi, 2017).

In respect to social outcomes, constructive STRs promote healthy outcomes such as self-concept, applying more social skills, and social-emotional adjustment (Baker, 2006). Furthermore, these relations reduce developmental vulnerabilities (Crosnoe et al., 2004), externalizing behavior outcomes (Silver et al., 2005) and social emotional problems (e.g., shyness, anxiety, school avoidance, and social withdrawal; Berry and O’Connor, 2010). Consequently, these positive relations lead to lower levels of aggression (Hughes et al., 2008), less discipline problems (Sáez et al., 2012), and greater level of students’ subjective wellbeing (Suldo et al., 2014). More recent evidence (Lan and Moscardino, 2019) showed that there is a positive connection between STR and student wellbeing including learning engagement, satisfaction with peer relations, and school satisfaction. In addition, the sense of security is provided when teachers establish a positive STRs, which enables students to take risks freely in the classroom, explore the social environment, admit mistakes, and ask for help (Davis, 2001). In contrast, negative relations are associated with external behavioral problems and internal symptoms such as anxiety (Baker, 2006). It is found that students who had poor STRs are more likely to have lower scores on social and emotional adjustment (either self or teacher-rated) compared with students who had positive relations (Murray and Greenberg, 2001). These students avoided class, negotiated the system, dropped out of school and had more behavior problems than their peers who have good relations (Fredricks et al., 2004).

#### Theoretical Orientations Examining the Student–Teacher Relationship

**FIGURE 1:**
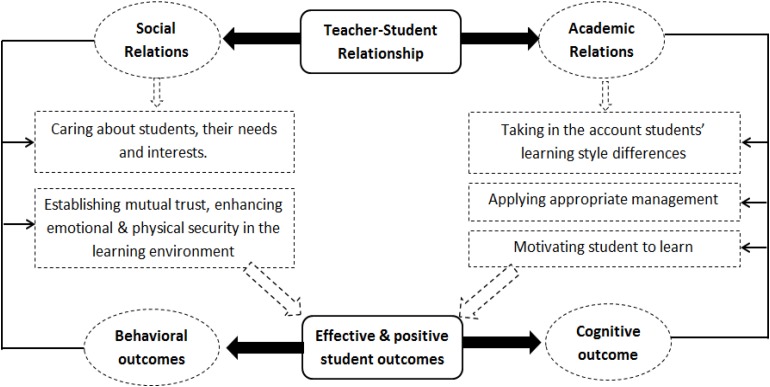
A theoretical model of the teacher–student relationship.

Many theoretical orientations have studied STR, including the Attachment theory, social cognitive theory, socio-cultural theory, ecological theory, and developmental systems theory. Corresponding, many dimensions have been examined in this relationship. Northup (2011) indicates three dimensions (i.e., satisfaction, instrumental help, and lack of conflict). Pianta (2001) shows other three dimensions (i.e., conflict, closeness and dependency). Moreover, Cranley-Gallagher and Mayer (2006) identifies four dimensions, which are recognition, familiarity, respect and commitment. Riddle (2003) points other four dimensions (i.e., trust, respect, communication, and discipline). In the current study, a new theoretical model has been proposed in which the STR consists of two dimensions. These are (1) Academic relations (AR) in terms of teacher instructional characteristics, and (2) Social relations (SR) in term of teacher personal characteristics, as shown in the model developed for this study (see Figure 1). According to the model, teachers establish positive AR by three tasks, which are: (1) Considering students’ learning style differences, (2) Applying an appropriate management style in the classroom, and (3) Motivating students to learn. On the other hand, teachers construct SR by: (1) Caring about students’ need and interests, (2) Establishing mutual trust, and (3) Enhancing emotional and physical security in the learning environment. Correspondingly, the quality of AR and SR determines the productiveness of students’ cognitive and behavioral/social outcomes.

The justification of the proposed model is articulated in a sequential order (i.e., from top to down), which includes: (1) Rationale of the two dimensions (AR and SR), (2) Intermediate instructional and social practices that teachers could implement to fulfill students’ needs, leading to positive relations, and (3) The association between constructive AR, SR and students’ outcomes. That is, the following review articulates several substantial supporting evidences, both theoretical and empirical.

The rationale of two dimensions has a root in two processes that require the teacher’s direct involvement and engagement with students, which are: an instruction from an academic perspective, and an interaction from a social perspective. Related to the first process, the teacher ensures higher quality of instruction by establishing well-tailored academic relationship with each student. In order to develop a fruitful AR, which is one dimension of the proposed model, teachers offer rich learning opportunities; provide support to scaffold students’ participation; demonstrate adequate academic expectations; enforce classroom norms; and design dynamic activities (Gregorg et al., 2014). Stronge et al. (2008) found that effective teachers who practice supportive instructional practices in four categories (i.e., instruction, student assessment, classroom management, and personal qualities) resulted in higher students’ learning gains among a sample of 1,936 students compared to ineffective teachers. A more recent study demonstrated that teacher practices (i.e., judgment and negative treatment) negatively associated with students’ future achievement, expectancy for success and level of aspiration (Zhu et al., 2018).

In respect with second process (i.e., interaction), teachers encourage students; show understanding and patience; promote positive recognition and rewards; make efforts to strengthen their relations with students (Schmakel, 2008); create a classroom environment that exhibits respect, care and trust between them and their students; and set high standards and expectations for better behavioral outcomes (Caballero, 2010). In addition, caring teachers interact with students informally; express a personal interest in them; respect their needs, interests and concerns; and demonstrate a commitment to their learning (Jepson, 2005). This results in constructive SR, which is another complimentary dimension of proposed model. A meta-analysis study (*n* = 57 studies from 2000 to 2016) showed a negative association between affective STRs and social outcome particularly externalizing behavior problems (Lei et al., 2016).

Correspondingly, the intermediate part of the current theoretical model specifies the five fundamental instructional and social practices that can fulfill students’ three basic psychological needs as identified in self-determination theory (Ryan and Deci, 2000). These needs are: (1) Relatedness (i.e., caring for and expressing interest), (2) Competence (i.e., setting clear rules and having consistent consequences), and (3) Autonomy (i.e., freedom given to students to make their own choices; and forging links between schoolwork and students’ interests; Roorda et al., 2011). That is, these needs can be fulfilled with the five practices (i.e., considering students’ learning styles; applying appropriate classroom management styles; caring about students’ needs and interests; establishing mutual trust; and promoting emotional and physical security in the learning environment).

The last part of the model suggests that teachers’ efforts in developing the high quality relations lead to effective and positive behavioral and cognitive student outcomes. Meaning, teacher’s positive AR and SR reinforce students’ developmental needs from a social, emotional and cognitive perspectives (Pianta et al., 2012; Gregorg et al., 2014) as supported by many theoretical frameworks. First, Attachment theory (Bowlby, 1969) implies that forming a relationship with one caring adult at the least is an important factor in protecting young students who are threatened by multiple risk factors in their lives, and this adult is usually the teacher (Sabol and Pianta, 2012). Lending further support is Vygotsky (1978), whose socio-cultural theory backs up this point through its focus on “scaffolding,” which refers to an individual with more developed skills (teacher) assisting an individual with less developed skills (student). In addition, Bronfenbrenner (1994) focuses on the principle of growth within the context of one’s environment. Or more specifically - the way in which the student grows through dynamic interaction with their immediate environment in different situations; and how this contributes to the formation of individual perceptions and beliefs, on four levels (microsystem, mesosystem, exosystem, and macrosystem). The teacher, according to Bronfenbrenner, belongs to the microsystem (the closest system), and this means that STRs have a considerable effect on students’ lives; a finding which is compatible with the current proposed model. Further agreement with the proposed model comes from Pianta (2001), who explains that supportive relations protect at-risk students from failure in school, and life in general. Pianta argues that teachers, in their caring and supportive relationship with students, belong to a dyadic system, a system that includes close interaction between the students and their parents, peers, and teachers, which can positively affect students’ outcomes.

Lastly, Social Cognitive Theory (SCT) demonstrates the significance of STRs by focusing on the triple impact of the individuals themselves, contextual influences, and behavior. In other words, an individual’s (student) behavior is a result of environmental influences (e.g., teacher and school environment) in light of the individual’s cognitive, intellectual and personal systems (Bandura, 1986). If we link SCT to the proposed STR model in the current study, teachers, unsurprisingly, appear to be one of the main components of school environment. They provide students with stimuli by applying different instructional strategies and allowing the practicing of daily social interaction. In other words, STR, both in academic terms (e.g., offering learning stimuli and activities, using diverse teaching styles, and specific instructional strategies) or social terms (e.g., using appropriate interaction styles; and building social relations based on understanding, trust, respect and safety) plays a major role in achieving positive students’ outcomes (Suleiman, 2010).

### Research Problem

As discussed earlier, STRs can be assessed using several approaches (i.e., assessing teachers’ perspectives, students’ perspectives, observations and case studies). In respect to teachers’ perspective, two key measures have identified. The first measure is the student–teacher relationship scale (STRS), which assesses the relationships with pre-school through up to 3rd grade students (Pianta, 2001). STRS includes 28 items that designed to measure three dimensions: Closeness, Conflict and Dependency. It has been noticed that the findings of the factor structure of this scale have changed according to the variations of the cultural contexts of different countries. For instance, some studies found that the results confirmed the 28 original items through using Confirmatory Factor Analysis (CFA; Glüer and Gregoriadis, 2016), while some other studies conducted in USA and Greece evaluated this scale and the findings did not show a satisfactory fit (Drugli and Hjemdal, 2012; Tsigilis et al., 2018). Thus, there are inconsistent findings of the factorial validity of this scale which emphasize the need to conduct further studies to evaluate the validity of STRS in different cultural backgrounds. The second measure is the teacher–student relationship inventory (TSRI), which measures the relationships with students in 4th grade up to junior school (Ang, 2005).

On the other hand, if the focus is to be on students, there are many measures that focus on emotional facets of relationships between teachers and junior, elementary, and middle school students (Lee, 2012). Some examples of these include the emotional quality scale of the relatedness questionnaire (Lynch and Cicchetti, 1997), the quality of teacher-student relationship scale (Davis, 2001); the network of relationships inventory (Meehan et al., 2003); and the inventory of teacher-student relationships (IT-SR; Murray and Zvoch, 2011). STRs have typically been measured as a sub-dimension involved in larger scales of social support (Malecki and Demaray, 2002) or as a single dimension based on items extracted from other scales (Ryan and Patrick, 2001).

Students look at the STR differently according to their age and grade levels. Students from 7 to 11th grades in middle and high schools are adolescents. This implies the presence of differences in their perspectives to STRs compared with younger students in elementary school (Wentzel, 1997; Lee, 2012). The literature has suggested that teenagers depend on their teachers for emotional support in different ways from younger students because they are more likely to seek challenges. Therefore, adult like learning activities combined with appropriate scaffolding may be the appropriate choice for teens (Wentzel, 1997; Gregorg et al., 2014). Moreover, the nature of relationships varies across grade levels (Northup, 2011). For example, middle school students rated good teachers as teachers who like them, help them, listen to them, and provide them with extra help (Kinney, 2007). Furthermore, good middle school teachers develop a strong relationship with their students, enhance their students’ desire for learning, and are interested in the success of all students (Koomen et al., 2012). They are also, supportive, non-judgmental and equal treatment provider to each student (Seaton, 2007). High school students, however, generally describe a good teacher as someone who has high expectations for their students, offers encouragement, builds good student relationships, and provides demanding learning tasks (Northup, 2011). Garza (2009) also adds that effective high school educators provide scaffolding during classroom lessons; have kind dispositions; are available to students when needed; are interested in students; show good manners; and promote wellbeing inside and outside the classroom.

In light of the aforementioned points, it has been noticed that many STRs scales measure the relation from a teacher’s perspective (Pianta, 2001; Ang, 2005). Most recent studies in this area examine very young children. Consequently, little is known about middle school students’ relationships with their teachers (Saft and Pianta, 2001) and seemingly even less about high school age students (Northup, 2011). To date, no standardized Arabic teacher–student relationship measure is available. In addition, many measurements for middle school students are only sub- dimensions of emotional measurements (Lee, 2012). Thus, little is known about STRs from students’ perspectives. The differences in students’ developmental characteristics and psychological needs of students in 7th-11th grades compared with younger students in elementary school require the use of a specific measure tailored for each group. The research goals are therefore:

• The development of a student- teacher relationship measure (S-TRM) for Omani students (7th–11th grades).
• An examination of the psychometric properties of the S-TRM through Exploratory Factor Analysis (EFA; study 1).
• Cross validation of the results of the S-TRM through CFA (study 2).
• An examination of the internal consistency coefficients and external validity for the measure and its’ dimensions and establishing evidences of external validity.

Thus, this study sought to answer the following question:

1- What are the psychometric properties of the student –teacher relationship measure (S-TRM) for Omani 7th-11th grades students?

Correspondingly, the current study hypothesized a two-factor solution (i.e., AR and SR) that load in a higher second-factor (i.e., STR). The scale was expected to have good psychometric properties including factorial structure and reliability. Furthermore, it hypothesized that the measure’s external validity is substantiated by: (1) Significant negative association with age and (2) Significant differences among grades in the quality of STR. That is, lower grades should have stronger STR relative to higher grades.

## Methodology

### Study Population and Sample

The population of this study was Omani female students from 7th to 11th grades, studying in public schools in one, large, and rural governorate in Oman. These students rated their relations with science teachers. The sample was selected for the following reasons. Examining the quality of relations between middle schools’ students and their science teachers is a prime concern in Oman by establishing sounded and abbreviated S-TRM scale. That is, Omani Ministry of Education aims to prepare students for the 4th Industrial Revolution (Al Harthy, 2019; Al-Rubaie, 2019). Yet, international tests (e.g., TIMSS) showed that 8th grade students scored (*M* = 455) in science, placing Oman at the moderate level as it is the case with other developing countries in the Middle East (Martin et al., 2016). This position is out of alignment with 4th Industrial Revolution requirements, raising a valid concern. As well, despite Omani females had higher science scores in these international tests relative to males (e.g., TIMSS; Martin et al., 2016), the literature has illustrated that females hold lower science identity (Hill et al., 2018). Furthermore, the selection of this rural governorate was owing to two reasons. First, it is large governorate that contain more than 36 female middle schools. Second, it assimilates other Omani rural governorates, covering diverse geographical regions. Data were collected randomly. A sample of female students (*n* = 2,134) was obtained. The sample of Study 1 covers four grades, which were: 7th (*n* = 284, 27.4%), 8th (*n* = 223, 21.5%), 9th (*n* = 245, 23.7%), 10th grade (*n* = 195, 18.8%), and 11th grade (*n* = 8.5%). Comparatively, Study 2 sample consist of 7th (*n* = 297), 8th (*n* = 250), 9th (*n* = 234), 10th (*n* = 214), and 11th (*n* = 104) grades.

### Procedure

First, the educational discrete general director typically grants permission for conducting research and data collection. The ethical committee in the general director granted ethical approval of the current study after going through a comprehensive process that involved submission of all related materials that ensured that the study would take place in accordance to the relevant ethics standards approved by the Ministry of Education Technical Office of Research and Development. Then, the general director sent official letters with a description of the current study. These letters explained the goals and procedure to all schools’ principles, and contained an attached invitation to attend a preparatory meeting to explain the study in depth for voluntary teachers. Later on, each selected teacher explained the study purpose to the students and received their consent to take part in the study. Additionally, parental consent forms were sent home for parents’ approval of their children’s participation in the study. Participation was strictly voluntary, and measure responses were kept confidential. The teachers and students were all informed that they could refuse or discontinue participation at any time. Approximately 2,200 copies of the questionnaire were administrated in Arabic because it is the main language used in the instruction for all Omani government schools. A total of 2,134 completed forms were collected with a return percentage of (97%).

### Measure

#### S-TRM Description

The S-TRM was prepared after reviewing the relevant literature. The final version of the measure includes 25 items distributed over two dimensions (AR and SR). The AR dimension has 15 items while SR dimension has 10 items. Examples items for the AR dimension are as follows: “*My teacher expects me to participate effectively in the classroom*,” and “*My teacher shows remarkable enthusiasm during teaching the subject.*” Measure items for the SR include: “*My teacher listens to what I say*,” and “*My teacher makes me trust in myself, my ability and my talents.*” Through using a 5-point Likert scale (1 = Definitely does not apply; 2 = Applies little; 3 = Applies sometimes; 4 = Applies often; 5 = Definitely applies), students rated to what extent they agreed with each statement.

#### S-TRM Construction

The following procedures were used to develop the S-TRM:

• An extensive review of literature that addressed STR scales was undertaken. This review included examining the Teacher-Student Relationship Questionnaire (TSRQ; Caballero, 2010), the Student-Instructor Relationship Scale (SIRS), the Student-Teacher Relationship Scale (STRS; Pianta, 2001), the Teacher-Student Relationship Scale (Partin, 1996), the Psychological and Social Climate in the Classroom Scale, and the Classroom Interaction Measure. Also, many studies that investigate the association between the STR and other variables were reviewed (Caballero, 2010; Roorda et al., 2011).
• After analyzing previous measures and studies, a theoretical model was built (as seen in Figure 1). The model indicates the presence of two dimensions. Correspondingly, initial pool of items was constructed (*n* = 35 items) covering the two dimensions: AR (*n* = 20 items) and SR (*n* = 15 items).
• The measure was reviewed by an evaluating committee that has members from the Psychology Department, and the Curriculum and Instruction Department in the Collage of Education. The review prompted participants to express their opinions about items in terms of language accuracy, correspondence to the dimensions, and suitability for the Omani environment. It also generated suggestions from committee members on various aspects of the measure. It was decided that items would be kept if the committee agreement percentage was above 60% and that items would be deleted if their agreement percentage was less than 60%. Based on advice from the committee, three items were modified, and the total number of items for the trial was 31 items.
• The measure was given to an exploratory sample for pilot testing. Six items were deleted due to the negative associations between these items and corresponding dimensions. Thus, 25 items were included in the final form of the S-TRM.

#### Initial Reliability Estimates of the S-TRM

The scale had a high internal consistency reliability (Cronbach’s α = 0.92) in the current sample. Cronbach’s Alpha values were good for dimensions (AR Cronbach’s α = 0.90 and SR Cronbach’s α = 0.87).

### Data Analysis

The current research was divided into two stages, in which different sets of statistical analyses were used. The two datasets were cleaned using the Statistical Package for Social Sciences (SPSS) for Windows Version 24.0 before conducting the analyses (e.g., missing data, normality, outliers). In Study 1, EFA, particularly Principal Axis Factoring (PAF) was used using SPSS. To identify the type of rotation to use, large inter-correlation coefficients between factors (i.e., *r* > 0.32) suggested the use of an oblique rotation (Costello and Osborne, 2005). Otherwise, Varimax rotation is used when the inter-correlation coefficients between factors is relatively small. Multiple assumptions were assessed, which included: multicollinearity, singularity, sampling adequacy and presence of identity matrix. A correlation matrix should reflect appropriate correlation coefficients (i.e.,0.08 > *r* > 0.03) to ensure no concern about multicollinearity. Additionally, the determinant should be small (i.e., >0) to avoid singularity. Furthermore, KMO values of 0.80 and above reflect “Good” to “Great” sampling adequacy, values of 0.70 suggest fair sample adequacy, values of 0.60 to 0.50 implies moderate to bad sample adequacy (Pett et al., 2003). Lastly, a significant *p*-value for Bartlett’s Test indicates that the correlation matrix is not an identity matrix (Thompson, 2004). Multiple criteria were examined to determine the number of extracted factors including: Kaiser’s rule of eigenvalues greater than one, scree plots, and the Parallel Test (Patil et al., 2008). Coefficient (Cronbach’s) Alpha was used to estimate internal consistency reliability. Construct validity was examined by investigating Pearson correlation coefficient and ANOVA test.

In Study 2, two CFA models (i.e., first-order and second-order CFA) were conducted using Mplus 8 (Muthén and Muthén, 2017). Maximum Likelihood (MLR) with robust standard errors was adopted to estimate the model indices. Additional specification was conducting by restricting the item with highest regression weight to one (Gonzalez and Griffin, 2001). This assists in identifying the metric scale for the latent variables (Little et al., 2006). That is, restricting an item’s loading to one identifies the amount of variance associated with a one-unit increase of a constrained regression weight (Gonzalez and Griffin, 2001). Several Goodness-of-Fit (GoF) indices were examined to evaluate the model’s fit (Schumacker and Lomax, 2016). These indices include Chi-Square (i.e., or the ratio of Chi-Square divided by the degree of freedom [χ^2^/df]), the RMSEA (Root Mean Square Error of Approximation), the Standardized Root Mean Residual (SRMR), the CFI (Comparative Fit Index), and the TLI (Tucker Lewis Index). A model shows good fit using the following criteria: (1) a non-significant χ^2^, and (2) RMSEA and SRMR values ≤ 0.05, and (3) CFI and TLI ≥ 0.95. As well, more liberal criteria (CFI and TLI ≥ 0.90) is indication of acceptable model fit. Furthermore, statistically significant direct and indirect effects (*p* < 0.05) were interpreted.

Lastly, measurement invariance was assessed using several Multi-group CFA models (Schumacker and Lomax, 2016). Four levels of measurement invariance were examined in a sequential order (i.e., configural, metric, scalar, and strict invariance; Putnick and Bornstein, 2016). That is, configural invariance was established when the items loaded into same factors in the sub-groups. Metric invariance is established by having similar factors loadings for the sub-groups. Equal factor loadings and items’ intercepts supported the scalar invariance. Lastly, strict invariance is substantiated by equal factor loadings, items’ intercepts and measurement errors. The gradual testing of the successive levels of invariance depends on the results of the formal level. For instance, falling to support measurement invariance in the configural model results in stopping the process of examining higher levels (e.g., metric; Milfont and Fischer, 2010). At each level of measurement invariance testing, several indices were examined to identify whether the higher level of measurement invariance is attained. These indices include: (1) significant chi-square differences, (2) Δ CFI (i.e., ≤0.01), (3) Δ TLI (i.e., = 0), Δ RMSEA (i.e., ≤0.01), and (5) lower values of BIC and adjusted BIC (Meade et al., 2008; Wang and Wang, 2012).

## Findings

### Study 1 Findings

#### Descriptive Statistics, Correlations, and Assumptions Checking

Descriptive statistics (see Table 1) were examined in addition to outliers (*z* ± 2.58) for the 25 items and no outliers were detected. No normality issues were identified. Results indicated that EFA assumptions were met. That is, the inter-items correlations were below 0.80, meaning no multicollinearity. Furthermore, singularity was not problematic because the determinant values were too small (0.07). The KMO test (0.97) was great pertaining to sample adequacy (Pett et al., 2003). Bartlett’s Test of Sphericity was significant, indicating that the correlation matrix was not an identity matrix (χ^2^[300] = 112088.49, *p* < 0.001).

**TABLE 1:** Descriptive statistics for the student–teacher relationship measure items (*N* = 25).

**Items**	***M***	***SD***
My teacher listens to what I say.	4.22	0.98
My teacher excites me for the lesson at the beginning of the class.	3.75	1.14
My teacher strengthens my confidence in my ability and talents.	3.90	1.10
My teacher encourages me to ask questions about the subject.	3.86	1.06
My teacher provides practical implications about the taught lessons.	4.01	1.07
My teacher cares about my academic performance.	4.20	0.99
My teacher uses teaching methods that suit my interest.	3.65	1.15
My teacher uses a variety of methods that captivate my attention.	3.82	1.12
My teacher links subject’s topics with characters that matter to us.	3.55	1.16
My teacher asks interesting questions related to the subject.	4.08	1.00
My teacher expects me to participate effectively in the classroom.	4.15	0.92
My teacher encourages positive interaction between students.	4.02	1.04
My teacher makes me feel that I am able to solve difficult questions.	3.86	1.09
My teacher encourages good behavior in the class.	4.15	1.00
My teacher encourages me to ask about thing that I did not understand.	4.16	1.04
My teacher encourages me to be the best I can.	4.10	1.04
My teacher makes me feel proud when I achieve certain goals.	4.12	1.08
My teacher gives some hints to provide the right answer.	3.98	1.10
My teacher uses teaching methods that develop my ability to cooperate with others.	3.74	1.111
My teacher shows remarkable enthusiasm during teaching the subject.	3.81	1.17
My teacher believes in me and my potential.	3.72	1.11
My teacher gives students an opportunity to think before answering questions.	4.14	0.99
My teacher involves students to answer the questions that are asked by their peers.	3.88	1.08
My teacher develops my self-confidence.	3.81	1.16
My teacher encourages students to find more than one way to solve problems.	3.98	1.03

*All items had a range from 1 to 5.*

#### EFA Results

In study 1, Principal Axis Factoring (PAF) with Direct Oblimin rotation was conducted because Principle Component Analysis (PCA) is not a valid factor analysis (Osborne, 2015). Furthermore, the direct Oblimin rotation was used because the correlation between the factors was high (*r* = 0.77 > 0.32), as suggested by Osborne (2015). Three main criteria were examined to determine the number of extracted factors including: Kaiser’s criterion of eigenvalues greater than 1, scree plots, and the Parallel Test (Patil et al., 2008). Also, items should preferably load greater than.40 on the relevant factor (Field, 2009). Though, a minimum value of factor loading (0.32) were acceptable too (Costello and Osborne, 2005).

**FIGURE 2:**
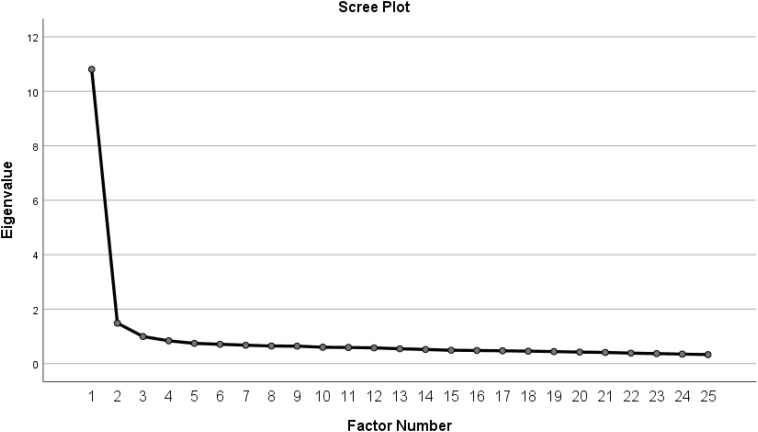
Scree plot of the teacher–student relationship measure.

The eigenvalues’ criterion, scree plot and Parallel Test demonstrated a two-factor solution (see Figure 2). All items in the pattern matrix loaded at.40 except two items. Items 19 and 20 had factor loadings greater than 0.32, meeting the minimum criteria identified by Costello and Osborne (2005). All 25 items had communalities greater than 0.30. Comparatively, the structure matrix revealed some cross loadings; however, the differences between loadings were relatively large (i.e., >0.150; Thompson, 2004). Thus, a final two-factor solution was retained, accounting for 49.15% of the variation (see Table 2). Factor 1 accounted for 43.21% of the variance and consisted of 15 items. Examples of significant items that loaded on this factor are: “My teacher gives some hints to help me reach the right answer” and “My teacher makes me feel that I am able to solve difficult questions.” This factor was labeled “Academic Relations” because it measures features of AR, in term of teachers’ instructional characteristics (e.g., the degree to which the teacher considers the differences in students’ learning styles, applies management styles, and motivates students).

**TABLE 2:** Loading factor with PAF with direct Oblimin (δ = 0) for the S-TRM.

	**Items**	**Factor 1: Academic Relations**	**Factor 2: Social Relations**
AR_LS_18	My teacher gives some hints to provide the right answer.	0.69	
AR_ML _24	My teacher develops my self-confidence.	0.68	
AR_ML_17	My teacher makes me feel proud when I achieve certain goals.	0.67	
AR_CM_16	My teacher encourages me to be the best I can.	0.66	
AR_ML _13	My teacher makes me feel that I am able to solve difficult questions.	0.66	
AR_CM_12.	My teacher encourages positive interaction between students.	0.64	
AR_ML _21	My teacher believes in me and my potential.	0.64	
AR_ML _15	My teacher encourages me to ask about thing that I did not understand.	0.64	
AR_CM_14	My teacher encourages good behavior in the class.	0.63	
AR_LS_23	My teacher involves students to answer the questions that are asked by their peers.	0.60	
AR_CM_11	My teacher expects me to participate effectively in the classroom.	0.59	
AR_LS_22	My teacher gives students an opportunity to think before answering questions.	0.55	
AR_LS_25	My teacher encourages students to find more than one way to solve problems.	0.49	
AR_ML_20	My teacher shows remarkable enthusiasm during teaching the subject.	0.39	
AR_LS_19	My teacher uses teaching methods that develop my ability to cooperate with others.	0.36	
SR_TRU_2	My teacher excites me for the lesson at the beginning of the class.		−0.78
SR_CAR_8	My teacher uses a variety of methods that captivate my attention.		−0.77
SR_TRU_7	My teacher uses teaching methods that suit my interest.		−0.76
SR_CAR_5	My teacher provides practical implications about the taught lessons.		−0.70
SR_TRU_3	My teacher strengthens my confidence in my ability and talents.		−0.65
SR_TRU_10	My teacher asks interesting questions related to the subject.		−0.65
SR_CAR_9	My teacher links subject’s topics with characters that matter to us.		−0.63
SR_TRU _4	My teacher encourages me to ask questions about the subject.		−0.54
SR_TRU_1	My teacher listens to what I say.		−0.45
SR_CAR_6	My teacher cares about my academic performance.		−0.41
	Cronbach’s alpha	0.92	0.89
	Omega	0.92 [0.91–0.93]	0.91 [0.91–0.92]
	Eigenvalue	10.80	1.48
	Variance explained	43.21%	5.94%

Factor 2 accounted for 5.94% of the variance. Ten items loaded in this factor (e.g., “My teacher makes me excited about the lesson at the beginning of the class” and “My teacher makes me trust in myself, my ability and my talents”). This factor aligns with “Social Relations,” reflecting aspects of the SR in term of teachers’ personal characteristics (e.g., level of caring, and ability to promote trust and provide a safe learning environment). Internal consistency estimates were computed using Cronbach’s alpha coefficients. AR had a good reliability (Cronbach’s α = 0.92, 95% CI [0.91−0.93]). Also, the reliability of the SR was good (i.e., Cronbach’s α = 0.89, 95% CI [0.89−0.90]). The reliability for the total measure was α = 0.95 (*N* = 25 items, 95% CI [0.94−0.95]). Omega coefficients were good (see Table 2).

#### Validity Indices

Validity is the process by which “a test developer or test user collects evidence to support the types of inferences that are to be drawn from test scores” (Crocker and Algina, 2008, p. 217). In order to support the scale external validity, particularly construct validity was investigated. Literature has articulated that STR is associated negatively to students’ age (Lee, 2012). Meaning, the quality of STR decreases as students get older. A meta-analysis study (*n* = 65 studies from 1994 to 2016) revealed that age moderated the correlation between STR and academic emotions (i.e., either positive emotions [e.g., enjoyment, pride] and negative emotions [e.g., shame, anxiety]; Lei et al., 2018).

In current study, external validity can be supported by negative association between STR and age. Furthermore, establishing significant differences between grades should provide additional indicator of external validity. That is, the STR among lower grades (i.e., 7th and 8th grade) students should be stronger than relations among higher grades students (i.e., 9th, 10th, and 11th grade). Pearson correlation coefficients indicated that age negatively associated with SR (*r* = −0.25, *p* < 0.001), AR (*r* = −0.19, *p* < 0.001), and STR (*r* = −0.24, *p* < 0.001). One-way ANOVA investigated the STR differences between the lower and high grades. Examining the assumptions demonstrated that normality was met. Homogeneity of variance was violated among groups, suggested the interpretation of Brown-Forsythe values. Results illustrated significant differences between grades in the STR (see Table 3). Given the fact of unequal sample size between grades, Bonferroni test was conducted. *Post hoc* comparisons showed that lower grades (7th and 8th grades) had higher rates for their social, academic and overall STR compared with students in higher grades (10th and 11th grades), supporting construct validity. No significant differences were identified between 8th and 9th grades in the rating of the SR, AR, and STR.

**TABLE 3:** Student–teacher relationship differences across grades ANOVA results.

**Motives**	**Source**	**SS**	**df**	**MS**	**F**	**Effect Size (η^2^)**
Social Relation	Grade	43.74	4	20.94	18.67^∗∗∗^	0.07
	Within	572.34	1,030	0.56		
	Total	646.09	1,034			
Academic Relation	Use groups	27.64	4	6.91	13.27^∗∗∗^	0.05
	Within	519.53	1,030	0.50		
	Total	547.17	1,034			
STR	Use groups	34.61	4	8.65	17.81^∗∗∗^	0.07
	Within	478.31	1,030	0.46		
	Total	512.92	1,034			

*^∗∗∗^*p* < 0.001.*

### Study 2 Findings

Study 2 aimed to confirm the two-factor solution that was obtained in Study 1 through conducting two CFA models (i.e., first- and second-order CFA) using Mplus 8 among a new sample (*n* = 1, 099). First-order CFA results illustrated relatively a good model fit as supported by majority of fit indices expect Chi-square (see the upper part of Table 4). That is, the Chi-square GoF test (χ*^2^*[274] = 1055.69; *p* < 0.001) was significant, suggesting poor model fit. The significance of Chi-square can be attributed to large sample size (*n* = 1,099; Wang and Wang, 2012). However, The RMSEA was 0.05 (95% CI [0.048−0.054]). Comparatively, SRMR supported good model fit (0.03). CFI and TLI were 0.95, suggesting a good model fit. Standardized coefficients were statistically significant. Reviewing modification indices did not showed major changes that could enhance the Chi-square GoF test. Thus, no modifications terms (e.g., error covariances) were added, suggesting the acceptance of the initial model findings.

**TABLE 4:** Maximum likelihood standardized estimates and fit indices for the first-order and second-order CFA of the S-TRM.

**Fit Statistics**	**Factor 1: Academic Relations**	**Factor 2: Social Relations**	***R*^2^**

***Fit Indices for the First-order CFA of the Student –Teacher Relationship Measure***			
**Factor loadings**			
SR1. My teacher listens to what I say.		0.64^∗∗∗^	0.41^∗∗∗^
SR2. My teacher excites me for the lesson at the beginning of the class.		0.77^∗∗∗^	0.59^∗∗∗^
SR3. My teacher strengthens my confidence in my ability and talents.		0.77^∗∗∗^	0.60^∗∗∗^
SR4. My teacher encourages me to ask questions about the subject.		0.70^∗∗∗^	0.48^∗∗∗^
SR5. My teacher provides practical implications about the taught lessons.		0.76^∗∗∗^	0.58^∗∗∗^
SR6. My teacher cares about my academic performance.		0.66^∗∗∗^	0.44^∗∗∗^
SR7. My teacher uses teaching methods that suit my interest.		0.79^∗∗∗^	0.62^∗∗∗^
SR8. My teacher uses a variety of methods that captivate my attention.		0.75^∗∗∗^	0.56^∗∗∗^
SR9. My teacher links subject’s topics with characters that matter to us.		0.66^∗∗∗^	0.43^∗∗∗^
SR10. My teacher asks interesting questions related to the subject.		0.74^∗∗∗^	0.54^∗∗∗^
AR11. My teacher expects me to participate effectively in the classroom.	0.68^∗∗∗^		0.47^∗∗∗^
AR12. My teacher encourages positive interaction between students.	0.72^∗∗∗^		0.52^∗∗∗^
AR13. My teacher makes me feel that I am able to solve difficult questions.	0.74^∗∗∗^		0.55^∗∗∗^
AR14. My teacher encourages good behavior in the class.	0.66^∗∗∗^		0.43^∗∗∗^
AR15. My teacher encourages me to ask about thing that I did not understand.	0.65^∗∗∗^		0.42^∗∗∗^
AR16. My teacher encourages me to be the best I can.	0.75^∗∗∗^		0.56^∗∗∗^
AR17. My teacher makes me feel proud when I achieve certain goals.	0.71^∗∗∗^		0.50^∗∗∗^
AR18. My teacher gives some hints to provide the right answer.	0.63^∗∗∗^		0.39^∗∗∗^
AR19. My teacher uses teaching methods that develop my ability to cooperate with others.	0.76^∗∗∗^		0.58^∗∗∗^
AR20. My teacher shows remarkable enthusiasm during teaching the subject.	0.75^∗∗∗^		0.56^∗∗∗^
AR21. My teacher believes in me and my potential.	0.77^∗∗∗^		0.59^∗∗∗^
AR22. My teacher gives students an opportunity to think before answering questions.	0.65^∗∗∗^		0.42^∗∗∗^
AR23. My teacher involves students to answer the questions that are asked by their peers.	0.56^∗∗∗^		0.32^∗∗∗^
AR24. My teacher develops my self-confidence.	0.77^∗∗∗^		0.60^∗∗∗^
AR25. My teacher encourages students to find more than one way to solve problems.	0.72^∗∗∗^		0.52^∗∗∗^
**Fit Indices**			
χ^2^(*df*)	1055.69(274)		
*p*-value	0.00		
RMSEA	0.05, 95% CI [0.048–0.054]		
SRMR	0.03		
GFI	0.95		
NFI	0.95		

***Fit Indices for the Second-order CFA of the Student –Teacher Relationship Measure***			
	**General Factor: Student–Teacher Relationship**		***R*^2^**

**Factor loadings**			
Academic Relation (AR)	0.92^∗∗∗^		0.84^∗∗∗^
Academic Relation (SR)	0.99^∗∗∗^		0.98^∗∗∗^
**Fit Indices**			
χ^2^(*df*)	706.81(273)		
*p*-value	0		
RMSEA	0.04, 95% CI [0.035–0.041]		
SRMR	0.03		
CFI	0.96		
TLI	0.96		

*^∗∗∗^*p* < 0.001.*

**FIGURE 3:**
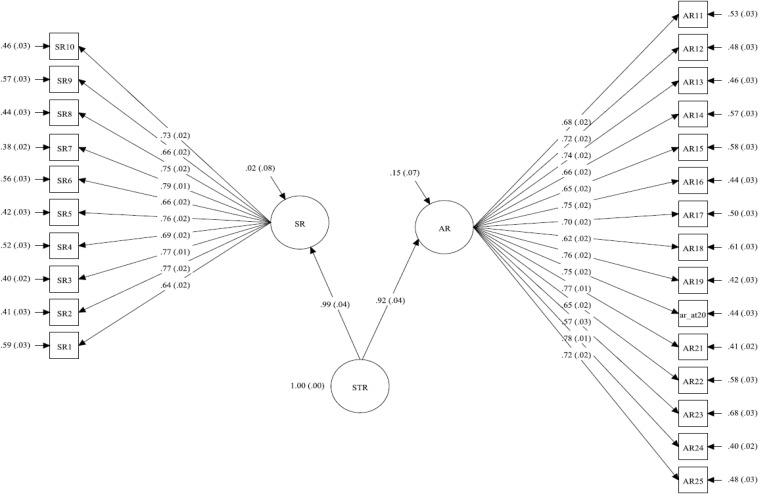
The standardized estimates of second-order student–teacher relationship measure.

The findings suggest that the two factors (i.e., SR and AR) can be loaded into general factor. Bifactor or second-order CFA models were candidate to appropriately model this general factor. The association between sub-dimensions and the general factor identified the most suitable model. As stated by Rodriguez et al. (2016) stated that “Commonly assumed, too, is that the general and group factors are orthogonal” (p. 137) in bifactor model. Meaning, bifactor model should be used when the group factors (sub-dimensions) and the general factor are uncorrelated. In this study, the sub-dimensions (i.e., SR and AR) are correlated with the general factor (i.e., STR). Thus, second-order CFA model is more appropriate. However, in a preliminary step, an exploratory bi-factor analysis was conducted to assess the unidimensionality of the scale. According to Jennrich and Bentler (2011), identifies the degree to which the group factors account for the departure from unidimensionality. In particular, Mplus 8 was used to conduct exploratory bi-factor analysis with bi-geomin rotation (i.e., oblique rotation), considering the significant association between AR, SR and STR (Muthén and Muthén, 2017). Findings showed good model fit as implied by four fit indices (RMSEA, SRMR, CFI and TLI were.04,0.02,0.97, and 0.96 respectively), supporting the multidimensionality of the scale. In contrast, significant Chi-square GoF test (χ*^2^*[251] = 670.50; *p* < 0.001) suggested poor model fit. In the second step, the second-order CFA model was conducted. Chi-square GoF test (χ*^2^*[273] = 706.81; *p* < 0.001) was significant, suggesting poor model fit that can be justified by this test’s sensitivity to large sample size (Wang and Wang, 2012). On the other hand, RMSEA, SRMR, CFI and TLI demonstrated good model fit (see the bottom part of Table 4). In details, the RMSEA and SRMR were small (i.e., 0.04, 95% CI [0.035−0.04], and 0.03, respectively). CFI and TLI were >0.95. The standardized coefficients were statistically significant (see Figure 3). Reviewing modification indices showed no substantive modifications, suggesting the acceptance of second-order CFA model.

In order to access scale’ measurement invariance, two grades were selected, which were lower (i.e., 7th grade) and higher grades (i.e., 9th grade), representing relatively middle and high school. In a preliminary step, two baseline CFA models were fitted to 7th grade (*n* = 297) and 9th grade (*n* = 234) samples. For the 7th grade sample, findings suggested good model fit as supported by four fit indices (RMSEA, SRMR, CFI and TLI were.05, 95% CI [0.041−0.056]0.04, 0.95, and 0.94 respectively). Though, Chi-square GoF test (χ^2^[274] = 467.83; *p* < 0.001) was significant. For the 9th grade, findings showed poor initial model fit as implied by the majority of fit indices. Modification indices proposed the addition of two error covariance (i.e., AR14 with AR11 and AR21 with AR20). The model had an acceptable fit after adding the suggested error covariance terms. That is, RMSEA, SRMR, CFI and TLI were.07, 95% CI [0.057–0.073]0.05, 0.92, and 0.91 respectively). These differences imply some preliminary concerns about the measurement invariance.

In respect to measurement invariance, only configural and metric invariance were examined by fitting the Multi-group CFA model (see Table 5). Results of configural invariance significant Chi-square tests, whereas RMSEA, SRMR, GFI, TLI supported configural invariance between 7th and 9th grades (i.e.,0.05, 95% CI [0.051–0.062]0.04, 0.93, and 0.93 respectively). Establishing evidence of configural invariance supported the investigation of the next higher level of invariance (i.e., metric invariance; Milfont and Fischer, 2010). Findings of metric invariance showed (1) significant chi-square differences between configural (Model 1) and metric invariance (Model2), (2) higher value of RMSEA, (3) unacceptable SRMR, (4) no differences between CFI (Δ CFI = 0), (6) lower value of TLI (Δ TLI = 0.01) and BIC, and (7) higher value of adjusted BIC. Decision related to accepting the metric invariance was identified based on the multiple criteria (Meade et al., 2008; Wang and Wang, 2012). That is, metric invariance is rejected because of large value of SRMR (i.e., >0.05), larger value of adjusted BIC and RMSEA, and lower value of TLI. Though, the differences in RMSEA and TLI were in the acceptable range (i.e., ≤0.01). Overall, metric measurement invariance was not supported leading to discontinuity of measurement invariance assessment as suggested by Putnick and Bornstein (2016).

**TABLE 5:** Comparison of two levels of measurement invariance for STRM between lower (7th) and higher (9th) grades.

**Models**	**χ^2^(*df*), *p*-value**	**Δχ^2^ (Δ*df*)**	**RMSEA (90%CI)**	**SRMR**	**GFI (Δ GFI)**	**TLI (Δ TLI)**	**BIC**	**Adjusted BIC**	**Comparison**	**Decision**
Model 1: Configural invariance	1009.07(544), *p* < 0.001	–	0.05 (0.051–0.062)	0.04	0.93(−)	0.93(−)	32372.56	31877.37	–	Accepted
Model 2: Metric invariance	1076.02(548), *p* < 0.001	66.95(4)	0.06 (0.055–0.066)	0.20	0.93(0)	0.92(0.01)	31764.65	31931.92	Model 1 vs. Model 2	Rejected

## Discussion

The present study provides a new theoretical model for the STR. The results support the theoretical model and bi-dimensionality of the new developed measure (S-TRM). Overall, the two-factor model showed a good model fit with both EFA and CFA, and provided convincing evidence supporting the appropriateness of the S-TRM for use in further research. The preliminary version of the measure was developed with 35 items. After finalizing arbitration procedures and analyzing exploratory sample results, the number of items was reduced to 25 items. In Study 1, a sample of 1,035 students was tested using EFA, which revealed two factors. These factors were labeled academic relations (AR) and social relations (SR). Both factors were moderately correlated with each other. Evidence of measure’s external validity was supported by significant negative association between age, SR, AR, and STR. ANOVA findings showed significant differences between lower (i.e., 7th and 8th) and higher grades (i.e., 10th and 11th), providing an additional evidence of external validity. Further analysis in Study 2 was conducted using two CFA models with a new sample to validate the hypothesized two-factor model. Findings endorsed the hypothesized two-factor model convincingly; meeting four CFA criteria and indices. As well, scale dimensionality and measurement invariance across lower and higher grades were assessed. In effect, three major results are presented: The existence of two factors (i.e., SR and AR), the existence of general factor (i.e., STR), and the establishment of configural measurement invariance across lower and higher grades.

The first result is compatible with empirical results (Schmakel, 2008; Caballero, 2010; Camp, 2011; Roorda et al., 2011; Gregorg et al., 2014) that advocate the effect of both academic instruction and social interaction on students’ outcomes. On the other hand, this finding is inconsistent with dimensionality structure (Riddle, 2003; Cranley-Gallagher and Mayer, 2006; Scherzo, 2010; Northup, 2011). Such expected findings can be explained from both theoretical and procedural standpoints.

Theoretically, according to Vygotsky (1978), teachers scaffold students to help them gradually improve both their academic and social skills. Both Bandura (1986) and Bronfenbrenner (1994) indicate the importance of environmental stimuli on learning, specifically those stimuli that are very close to students, such as a teacher. Moreover, Attachment theory and Pianta’s (2001) developmental systems theory focus on the role of the STR, especially for at-risk students. Self-determination theory (Ryan and Deci, 2000) addresses three psychological needs that have to be fulfilled by the teacher relatedness, competence and autonomy so that positive STR has a significant impact on students’ need satisfactory (Bakadorova and Raufelder, 2018). Unsurprisingly, it has been shown that teachers play a major role in enhancing positive student’s academic performance by offering learning stimuli and activities, using different teaching styles and specific teaching techniques and learning strategies. Additionally, this is true as well on a personal/social basis where positive outcomes are promoted through using communication styles, and establishing strong social relations based on understanding, trust, respect and safety (Suleiman, 2010).

At a procedural level, the literature indicates the academic and social roles teachers have, and their obligations toward students. Academically, these include: contributing ample learning opportunities, and offering support through scaffolding students’ classroom participation (Gregorg et al., 2014); promoting students’ attention by offering interesting activities (Emmer and Stough, 2001); as well as demonstrating academic expectations, enforcing classroom norms, and designing dynamic activities (Gregorg et al., 2014). During their quest to meet these obligations, teachers apply many intermediate processes that require attention, variation, and development in an integrated manner. Among these processes are the teacher’s ability to take into consideration the adaptation of teaching methods based on students’ learning styles, the use of different classroom management styles, and the motivation provided for the students to learn. All these processes contribute to form positive academic relationships (Khazaaleh et al., 2011).

From a social/personal point of view, it is thought that teachers should motivate students, understand them and be patient, show positive recognition, provide rewards, and make great efforts to build a strong relationship with their students (Schmakel, 2008). Additionally, they should create a classroom climate that promotes mutual respect, strengthens the relationship between them and their students through enhancing a sense of care and trust and determines high standards and expectations in order to gain positive behavioral outcomes (Caballero, 2010). Other studies have mentioned the following actions as being indicative of a caring teacher: using an informal communication with students, considering students’ needs and interests, demonstrating interest and concern to teach and build a good teacher-student relationship, and showing commitment to students’ learning (Jepson, 2005). According to Camp (2011), effective teachers exhibit characteristics and utilize certain communication behaviors and styles which enable them to build positive social relationships. Among the behaviors as Camp (2011) mentioned are caring about students, and their needs and interests; striving to establish mutual trust, enhancing emotional and physical security; and creating a safe learning environment. Thus, both proposed AR and SR dimensions and their general factor (STR) are supported.

Meaningful differences between students in middle and high school should be examined after establishing evidences related to scale measurement invariance across four levels of invariance. The current study showed that only one level of invariance was substantiated (i.e., configural invariance) between 7th and 9th grades as representative grades of lower and higher grades in middle/high schools. On contrast, metric invariance was supported by only four fit indices (i.e., significant chi-square difference, acceptable Δ RMSEA, Δ TLI and lower BIC). In contrast, unacceptable SRMR, higher value of adjusted BIC and RMSEA, and lower value of TLI implied rejecting the metric invariance. These findings necessitated further investigation of measurement invariance, particularly metric, scalar and strong invariance across grades.

Due to the importance of STR, many significant implications can be suggested (1) Set up workshops and training sessions for teachers on the principles of establishing positive relationships with students, (2) Supply training courses in order to change education supervisors’ perspectives to focus on both teachers’ academic and social competences affecting students’ learning; and (3) Set up workshops and training sessions for teachers on the principles of establishing positive relationships with students.

A number of limitations of our research should be listed. Firstly, the sample consists only of female students in 7th–11th grades in Oman. Some studies found that positive, supportive, and less-conflict STRs are more perceived by female students than male students (Katz, 2017; Zee and Koomen, 2017). Secondly, students reported their perspectives about their relationships with science teachers only. Only, one level of measurement invariance was supported (i.e., configural invariance). Finally, we did not test the entire model in Figure 1.

Regarding further studies, the following is a list of recommendation for future research: (1) Compare factorial structure across gender and grades (i.e., assessing higher levels of measurement invariance, particularly metric, scalar and strong invariance); (2) Re-test psychometric properties of the (S-TRM) using different samples, and (3) Run experimental studies in order to examine the effects of training courses specialized to build positive STRs, and how they differ across different grades.

## Data Availability Statement

The datasets generated for this study are available on request to the corresponding author.

## Ethics Statement

The studies involving human participants were reviewed and approved by the Technical Office of Research and Development in the Ministry of Education in Oman. The patients/participants’ guardians provided written informed consent on behalf of the participants, to participate in this study.

## Author Contributions

Both authors have extensively participated in conducting everything in the research starting from identifying the problem, stating the research questions, reviewing the literature, identifying the population, developing the instrument, collecting data, and finishing with analyzing and interpreting the results.

## Conflict of Interest

The authors declare that the research was conducted in the absence of any commercial or financial relationships that could be construed as a potential conflict of interest.

## References

[B1] Al Harthy H. (2019). “The orientations of the ministry of education in the sultanate of Oman to keep up with the 4th industrial revolution,” in *Paper Presented at the International Conference of Fourth Industrial Revolution and its Impact on Education*, (Suhar).

[B2] Alhadabi A. (2013). *The Effectiveness of a Training Program to Science Teachers Based on Personality Type Theory in Developing Teacher-Student Relationship and Motivation to Learn Science of Grades (5-12) students in the Sultanate of Oman.* Master dissertation, Sultan Qaboos University , Muscat

[B3] Alrajhi M. Aldhafri S. (2015). Academic and social self-concept: effects of teaching styles and gender in English as a second language setting. *J. Psychol. Africa* 25 1–6.

[B4] Al-Rubaie S. (2019). “The orientations of education in Oman under the 4th industrial revolution,” in *Paper Presented at the International Conference of Fourth Industrial Revolution and its Impact on Education*, (Suhar).

[B5] Ang R. (2005). Development and validation of the teacher-students relationship inventory using exploratory and confirmatory factor analysis. *J. Exp. Educ.* 47 55–73.

[B6] Bakadorova O. Raufelder D. (2018). The essential role of the teacher-student relationship in students’ need satisfaction during adolescence. *J. Appl. Dev. Psychol.* 58 57–65. 10.1016/j.appdev.2018.08.004

[B7] Baker J. (2006). Contributions of teacher–child relationships to positive school adjustment during elementary school. *J. Sch. Psychol.* 44 211–229. 10.1016/j.jsp.2006.02.002

[B8] Baker J. Grant S. Morlock L. (2008). The teacher–student relationship as a developmental context for children with internalizing or externalizing behavior problems. *Sch. Psychol. Q.* 23 3–15. 10.1037/1045-3830.23.1.3

[B9] Bakhshaee F. Hejazi E. (2017). Student’s academic engagement: the relation between teacher’s academic optimism and female student’s perception of school climate. *Int. J. f Men. Health Addict.* 15 646–651. 10.1007/s11469-016-9674-2

[B10] Bandura A. (1986). *Social Foundations of Thought and Action: A social Cognitive Theory.* Englewood Cliffs, N.J: Prentice-Hall.

[B11] Berry D. O’Connor E. (2010). Behavioral risk, teacher–child relationships, and social skill development across middle childhood: a child-by-environment analysis of change. *J. Appl. Dev. Psychol.* 31 1–14. 10.1016/j.appdev.2009.05.001

[B12] Bowlby J. (1969). *Attachment and Loss.* New York, NY: Basic Books.

[B13] Brewster A. Bowen G. (2004). Teacher support and the school engagement of Latino middle and high school students at risk of school failure. *Child Adolesc. Soc. Work J.* 21 47–67. 10.1023/b:casw.0000012348.83939.6b

[B14] Bronfenbrenner U. (1994). Ecological models of human development. *Readings Dev. Child.* 2 37–43.

[B15] Caballero J. (2010). *The Effects of the Teacher-Student Relationship, Teacher Expectancy, and Culturally-Relevant Pedagogy on Student Academic Achievement.* Ph.D.Thesis, University of Redlands, Redlands, CA.

[B16] Camp M. (2011). *The Power of Teacher-Student Relationships in Determining Student Success.* Doctoral thesis, University of Missouri-Kansas City, Kansas City

[B17] Costello A. B. Osborne J. (2005). Practical assessment, research and evaluation. *J. Consum. Mark.* 10 1–9.

[B18] Cranley-Gallagher K. Mayer K. (2006). Teacher-child relationships at the forefront of effective practice. *Young Child.* 61 44–49.

[B19] Crocker L. M. Algina J. (2008). *Introduction to Classical and Modern Test Theory*. Mason, OH: Cengage Learning.

[B20] Crosnoe R. Johnson M. Elder G. (2004). Inter-generational bonding in school: the behavioral and contextual correlates of student–teacher relationships. *Sociol. Educ.* 77 60–81. 10.1177/003804070407700103

[B21] Davis H. (2001). The quality and impact of relationships between elementary school students and teachers. *Contemp. Educ. Psychol.* 26 431–453. 10.1006/ceps.2000.1068 11681827

[B22] Deiro J. (2003). Do your students know you care? *Educ. Leadersh.* 60 60–62.

[B23] DiLalla L. Marcus T. Wright-Phillips M. (2004). Longitudinal effects of preschool behavioral styles on early adolescent school performance. *J. Sch. Psychol.* 42 385–401. 10.1016/j.jsp.2004.05.002

[B24] Drugli M. Hjemdal O. (2012). Factor structure of the student–teacher relationship scale for Norwegian school-age children explored with confirmatory factor analysis. *Scand. J. Educ. Res.* 57 457–466. 10.1080/00313831.2012.656697

[B25] Emmer E. Stough L. (2001). Classroom management: a critical part of educational psychology, with implications for teacher education. *Educ. Psychol.* 36 103–112. 10.1207/s15326985ep3602_5

[B26] Engels M. Colpin H. Leeuwen K. Bijttebier P. Den Noortgate W. Claes S. (2016). Behavioral engagement, peer status, and teacher-student relationships in adolescence: a longitudinal study on reciprocal influences. *J. Youth Adolesc.* 45 1192–1207. 10.1007/s10964-016-0414-5 26759132

[B27] Field A. (2009). *Discovering Statistics Using SPSS.* London: SAGE Publications Ltd.

[B28] Fredricks J. Blumenfeld P. Paris A. (2004). School engagement: potential of the concept, state of the evidence. *Rev. Educ. Res.* 74 59–109. 10.3102/00346543074001059

[B29] Garza R. (2009). Latino and white high school students’ perceptions of caring behaviors: are we culturally responsive to our students? *Urban Educ.* 44 297–321. 10.1177/0042085908318714

[B30] Glüer M. Gregoriadis A. (2016). Quality of teacher–child relationship and preschoolers’ prosocial behaviour in German kindergartens. *Education* 3 1–14.

[B31] Gonzalez R. Griffin D. (2001). Testing parameters in structural equation modeling: every “one” matters. *Psychol. Methods* 6 258–269. 10.1037//1082-989x.6.3.258 11570231

[B32] Gregorg A. Allen J. Mikami A. Hafen C. Pianta R. (2014). Effects of a professional development program on behavioral engagement of students in middle and high school. *Psychol. Sch.* 51 143–163. 10.1002/pits.21741 28232767 PMC5319794

[B33] Hill P. Spiegel A. McQuillan J. Diamond J. (2018). Discovery orientation, cognitive schemas, and disparities in science identity in early adolescence. *Sociol. Perspect.* 61 99–125. 10.1177/0731121417724774 29576677 PMC5860849

[B34] Hofstede G. (2001). *Culture’s Consequences: Comparing Values, Behaviors, Institutions and Organizations Across Nations.* Thousand Oaks, CA: Sage.

[B35] Hughes J. Luo W. Kwok O. Lloyd L. (2008). Teacher–student support, effortful engagement and achievement: a 3-year longitudinal study. *J. Educ. Psychol.* 100 1–14. 10.1037/0022-0663.100.1.1 19578558 PMC2705122

[B36] Jennrich R. Bentler P. (2011). Exploratory bi-factor analysis. *Psychometrika* 76 537–549. 10.1007/s11336-011-9218-4 22232562 PMC3253271

[B37] Jepson C. (2005). Teacher characteristics and student achievement: evidence from teacher surveys. *J. Urban Econ.* 57 302–319. 10.1016/j.jue.2004.11.001

[B38] Katz I. (2017). In the eye of the beholder: motivational effects of gender differences in perceptions of teachers. *J. Exp. Educ.* 85 73–86. 10.1080/00220973.2015.1101533

[B39] Khazaaleh M. Al-Zabon M. Khazaaleh K. Shobaki A. (2011). *Effective teaching Methods.* Amman: Dar AL-Fikar.

[B40] Kinney P. (2007). A voice from the middle. *Principal Leaders.* 8 35–36.

[B41] Koomen H. Verschueren K. Schooten E. Jak S. Pianta R. (2012). Validating the student-teacher relationship scale: testing factor structure and measurement invariance across child gender and age in a Dutch sample. *J. Sch. Psychol.* 50 215–234. 10.1016/j.jsp.2011.09.001 22386121

[B42] Lan X. Moscardino U. (2019). Direct and interactive effects of perceived teacher-student relationship and grit on student wellbeing among stay-behind early adolescents in urban China. *Learn. Individ. Differ.* 69 129–137. 10.1016/j.lindif.2018.12.003

[B43] Lee R. (2012). The effects of the teacher–student relationship and academic press on student engagement and academic performance. *Int. J. Educ. Res.* 53 330–340. 10.1016/j.ijer.2012.04.006

[B44] Lei H. Cui Y. Chiu M. (2016). Affective teacher-student relationships and students’ externalizing behavior problems: a meta-analysis. *Front. Psychol.* 7:1311. 10.3389/fpsyg.2016.01311 27625624 PMC5003892

[B45] Lei H. Cui Y. Chiu M. (2018). The relationship between teacher support and students’ academic emotions: a meta-analysis. *Front. Psychol.* 8:2288. 10.3389/fpsyg.2017.02288 29403405 PMC5786576

[B46] Little T. Siegers D. Card N. (2006). A non-arbitrary method of identifying and scaling latent variables in SEM and MACS models. *Struct. Equ. Modeling* 13 59–72. 10.1207/s15328007sem1301_3

[B47] Lynch M. Cicchetti D. (1997). Children’s relationships with adults and peers: an examination of elementary and junior high school students. *J. Sch. Psychol.* 35 81–99. 10.1016/s0022-4405(96)00031-3

[B48] Malecki C. Demaray M. (2002). Measuring perceived social support: development of the child and adolescent social support scale (CASSS). *Psychol. Sch.* 39 1–18. 10.1002/pits.10004

[B49] Martin M. Mullis I. Foy P. Hooper M. (2016). *TIMSS 2015 International Results in Science.* Available at: http://timssandpirls.bc.edu/timss2015/international-results/wp-content/uploads/filebase/full%20pdfs/T15-International-Results-in-Science-Grade-4.pdf (accessed December 15, 2018).

[B50] Meade A. Johnson E. Braddy P. (2008). Power and sensitivity of alternative fit indices in tests of measurement invariance. *J. Appl. Psychol.* 93 568–592. 10.1037/0021-9010.93.3.568 18457487

[B51] Meehan B. Hughes J. Cavell T. (2003). Teacher–child relationships as compensatory resources for aggressive children. *Child Dev.* 74 1145–1157. 10.1111/1467-8624.00598 12938710

[B52] Merritt R. (2018). *Classroom Environment.* Available at: http://ezproxysrv.squ.edu.om:2048/login?url=http://search.ebscohost.com/login.aspx?direct=true&db=e0h&AN=28544146&site=eds-live&scope=site (accessed January 10, 2019).

[B53] Mikk J. Krips H. Saalik U. Kalk K. (2016). Relationships between student perception of teacher-student relations and PISA results in mathematics and science. *Int. J. Sci. Math Educ.* 14 1437–1454. 10.1007/s10763-015-96697

[B54] Milfont T. Fischer R. (2010). Testing measurement invariance across groups: applications in cross-cultural research. *Int. J. Psychol. Res.* 3 111–121.

[B55] Murray C. Greenberg T. (2001). Relationships with teachers and bonds with school: social emotional adjustment correlates for children with and without disabilities. *Psychol. Sch.* 38 25–41. 10.1002/1520-6807(200101)38:1<25::aid-pits4>3.0.co;2-c

[B56] Murray C. Zvoch K. (2011). The inventory of teacher-student relationships: factor structure, reliability, and validity among African American youth in low-income urban schools. *J. f Early Adolesc.* 31 493–525. 10.1177/0272431610366250

[B57] Muthén L. Muthén B. (2017). *Mplus User’s Guide*, 8th Edn Los Angeles, CA: Muthén & Muthén.

[B58] Northup J. (2011). *Teacher and Student Relationships and Student Outcomes.* Ph.D.Thesis, University of Colorado Denver, Denver.

[B59] Osborne J. (2015). What is rotating in exploratory factor analysis? *Pract. Assess. Res. Eval.* 20 1–7.

[B60] Partin R. (1996). *Classroom Teacher’s Survival Guide.* Englewood Cliffs: Prentice-Hall.

[B61] Patil V. Singh S. Mishra S. Donovan D. (2008). Efficient theory development and factor retention criteria: abandon the ‘eigenvalue greater than one’ criterion. *J. Bus. Res.* 61 162–170. 10.1016/j.jbusres.2007.05.008

[B62] Pett M. Lackey N. Sullivan J. (2003). *Making Sense of Factor Analysis: The use of Factor Analysis for Instrument Development in Health care Research.* Thousand Oaks, CA: Sage Publications, Inc.

[B63] Pianta R. (2001). *Student–Teacher Relationship Scale (STRS): Professional Manual.* Lutz: Psychological Assessment Resources.

[B64] Pianta R. Hamre B. Allen J. (2012). *Teacher-Student Relationships and Engagement: Conceptualizing, Measuring, and Improving the Capacity of Classroom Interactions.* New York, NY: Springer.

[B65] Poulou M. (2017). Social and emotional learning and teacher-student relationships: preschool teachers’ and students’ perceptions. *Early Child. Educ. J.* 45 427–435. 10.1007/s10643-016-0800-3

[B66] Putnick D. Bornstein M. (2016). Measurement invariance conventions and reporting: the state of the art and future directions for psychological research. *Devel. Rev.* 41 71–90. 10.1016/j.dr.2016.06.004 27942093 PMC5145197

[B67] Riddle M. (2003). “Okay, kid! don’t make me hurt you: negotiating student/teacher relationships in the tumultuous classroom,” in *Annual Meeting of the Conference on College Composition and Communication*, (New York, NY).

[B68] Rodriguez A. Reise S. Haviland M. (2016). Evaluating bifactor models: calculating and interpreting statistical indices. *Psychol. Methods* 21 137–150. 10.1037/met0000045 26523435

[B69] Rodriguez L. (2008). Teachers know you can do more: understanding how school cultures of success after urban high school students. *Educ. Policy* 22 758–780. 10.1177/0895904807307070

[B70] Roorda D. Koomen H. Spilt J. Oort F. (2011). The influence of affective teacher–student relationships on students’ school engagement and achievement: a meta-analytic approach. *Rev. Educ. Res.* 81 493–529. 10.3102/0034654311421793

[B71] Ryan A. Patrick H. (2001). The classroom social environment and changes in adolescents’ motivation and engagement during middle school. *Am. Educ. Res. J.* 38 437–460. 10.3102/00028312038002437

[B72] Ryan R. Deci E. (2000). Self-determination theory and the facilitation of intrinsic motivation, social development, and well-being. *Am. Psychol.* 55 68–78. 10.1037//0003-066x.55.1.68 11392867

[B73] Sabol T. Pianta R. (2012). Recent trends in research on teacher–child relationships. *Attach. Hum. Dev.* 14 213–231. 10.1080/14616734.2012.672262 22537521

[B74] Sáez L. Folsom J. Al Otaiba S. Schatschneider C. (2012). Relations among student attention behaviors, teacher practices, and beginning word reading skill. *J. Learn. Disabil.* 45 418–432. 10.1177/0022219411431243 22207616 PMC3328644

[B75] Saft E. Pianta R. (2001). Teachers’ perceptions of their relationships with students: effects of child age, gender, and ethnicity of teachers and children. *Sch. Psychol. Q.* 16 125–141. 10.1521/scpq.16.2.125.18698

[B76] Scherzo K. (2010). *The Role of Teacher-student Relationships in an Early Childhood Comprehensive Application of Behavior Analysis to Schooling (CABAS^®^) Setting*. New York, NY: Columbia University.

[B77] Schmakel P. (2008). Early adolescents’ perspective on motivation and achievement in academics. *Urban Educ.* 43 723–749. 10.1177/0042085907311831

[B78] Schumacker R. Lomax R. (2016). *A Beginner’s Guide to Structural Equation Modeling*, 4th Edn New York, NY: Taylor & Francis Groups.

[B79] Seaton E. (2007). “If teachers are good to you”: caring for rural girls in the classroom. *J. Res. Rural Educ.* 22 1–16.

[B80] Silver R. Measelle J. Essex M. Armstrong J. (2005). Trajectories of externalizing behavior problems in the classroom: contributions of child characteristics, family characteristics, and the teacher–child relationship during the school transition. *J. Sch. Psychol.* 43 39–60. 10.1016/j.jsp.2004.11.003

[B81] Skinner E. Furrer C. Marchand G. Kindermann T. (2008). Engagement and disaffection in the classroom: part of a larger motivational dynamic? *J. Educ. Psychol.* 100 765–781. 10.1037/a0012840

[B82] Stronge J. Ward T. Tucker P. Hindman J. (2008). What is the relationship between teacher quality and student achievement? An exploratory study. *J. Pers. Eval. Educ.* 20 165–184. 10.1007/s11092-008-9053-z

[B83] Suldo S. McMahan M. Chappel A. Bateman L. (2014). Evaluation of the teacher–student relationship inventory in american high school students. *J. Psycheduc. Assess.* 32 3–14. 10.1177/0734282913485212

[B84] Suleiman S. (2010). *Reading in School Psychology.* Cairo: Book World.

[B85] Thompson B. (2004). *Exploratory and Confirmatory Factor Analysis: Understanding Concepts and Applications.* Washington, D.C: American Psychological Association.

[B86] Tsigilis N. Gregoriadis A. Grammatikopoulos V. (2018). Evaluating the student-teacher relationship scale in the greek educational setting: an item parcelling perspective. *Res. Pap. Educ.* 33 500–512.

[B87] Vygotsky L. S. (1978). *Mind in Society: The Development of Higher Psychological Processes.* Cambridge, MA: Harvard University Press.

[B88] Wang Z. Wang N. (2012). Knowledge sharing, innovation and firm performance. *Expert Syst. Appl.* 39 8899–8908. 10.1016/j.eswa.2012.02.017

[B89] Wentzel K. (1997). Student motivation in middle school: the role of perceived pedagogical caring. *J. Educ. Psychol.* 89 411–419. 10.1037//0022-0663.89.3.411

[B90] Wentzel K. (2002). Are effective teachers like good parents? Teaching styles and student adjustment in early adolescence. *Child Dev.* 73 287–301. 10.1111/1467-8624.00406 14717258

[B91] Zee M. Koomen H. (2017). Similarities and dissimilarities between teachers’ and students’ relationship views in upper elementary school: the role of personal teacher and student attributes. *J. Sch. Psychol.* 64 43–60. 10.1016/j.jsp.2017.04.007 28735607

[B92] Zhu M. Urhahne D. Rubie-Davies C. (2018). The longitudinal effects of teacher judgement and different teacher treatment on students’ academic outcomes. *Educ Psychol.* 38 648–668. 10.1080/01443410.2017.1412399

